# Pioglitazone Reverses Markers of Islet Beta-Cell De-Differentiation in *db/db* Mice While Modulating Expression of Genes Controlling Inflammation and Browning in White Adipose Tissue from Insulin-Resistant Mice and Humans

**DOI:** 10.3390/biomedicines9091189

**Published:** 2021-09-10

**Authors:** J. Jason Collier, Heidi M. Batdorf, Kaelan L. Merrifield, Thomas M. Martin, Ursula White, Eric Ravussin, David H. Burk, Chris R. Cooley, Michael D. Karlstad, Susan J. Burke

**Affiliations:** 1 Laboratory of Islet Biology and Inflammation, Pennington Biomedical Research Center, Baton Rouge, LA 70808, USA; heidi.batdorf@pbrc.edu (H.M.B.); Kmerri5@uic.edu (K.L.M.); thomas.martin@pbrc.edu (T.M.M.); 2 Physiology of Human Adipose Tissue, Pennington Biomedical Research Center, Baton Rouge, LA 70808, USA; ursula.white@pbrc.edu; 3 Human Physiology, Pennington Biomedical Research Center, Baton Rouge, LA 70808, USA; eric.ravussin@pbrc.edu; 4 Cell Biology and Bioimaging Core, Pennington Biomedical Research Center, Baton Rouge, LA 70808, USA; david.burk@pbrc.edu; 5 Department of Surgery, Graduate School of Medicine, University of Tennessee Health Science Center, Knoxville, TN 37920, USA; cooley.35@wright.edu (C.R.C.); mkarlsta@utmck.edu (M.D.K.); 6 Laboratory of Immunogenetics, Pennington Biomedical Research Center, Baton Rouge, LA 70808, USA

**Keywords:** diabetes, inflammation, obesity, thiazolidinedione

## Abstract

Obesity, insulin resistance, and type 2 diabetes contribute to increased morbidity and mortality in humans. The *db/db* mouse is an important mouse model that displays many key features of the human disease. Herein, we used the drug pioglitazone, a thiazolidinedione with insulin-sensitizing properties, to investigate blood glucose levels, indicators of islet β-cell health and maturity, and gene expression in adipose tissue. Oral administration of pioglitazone lowered blood glucose levels in *db/db* mice with a corresponding increase in respiratory quotient, which indicates improved whole-body carbohydrate utilization. In addition, white adipose tissue from *db/db* mice and from humans treated with pioglitazone showed increased expression of glycerol kinase. Both *db/db* mice and humans given pioglitazone displayed increased expression of *UCP-1*, a marker typically associated with brown adipose tissue. Moreover, pancreatic β-cells from *db/db* mice treated with pioglitazone had greater expression of insulin and Nkx6.1 as well as reduced abundance of the de-differentiation marker Aldh1a3. Collectively, these findings indicate that four weeks of pioglitazone therapy improved overall metabolic health in *db/db* mice. Our data are consistent with published reports of human subjects administered pioglitazone and with analysis of human adipose tissue taken from subjects treated with pioglitazone. In conclusion, the current study provides evidence that pioglitazone restores key markers of metabolic health and also showcases the utility of the *db/db* mouse to understand mechanisms associated with human metabolic disease and interventions that provide therapeutic benefit.

## 1. Introduction

Obesity and insulin resistance are predictors of the development of type 2 diabetes (T2D) [1,2,3]. Importantly, the progression to T2D requires the loss of islet β-cell mass, function, or both [4,5,6]. Strategies to protect total islet β-cell mass, insulin production, and insulin secretion are therefore sought to prevent onset of such metabolic diseases. Both pharmacological and lifestyle interventions can be successful at preventing or restoring metabolic tissue function to combat onset of hyperglycemia, a critical defining feature of T2D [7,8]. Lifestyle interventions typically target weight reduction, leading to decreases in tissue lipid content that restore organ function [7,9]. Alternatively, weight reduction is not typically required for the therapeutic effects of many pharmaceutical approaches, such as administration of metformin or thiazolidinediones (TZDs).

FDA-approved TZDs, such as rosiglitazone and pioglitazone, often promote weight gain despite strong insulin-sensitizing properties. However, this weight gain appears to be preferentially in subcutaneous regions, which likely contributes to the improved metabolic health despite increased total fat mass [10]. Indeed, the power of TZDs to prevent progression to T2D was revealed in several clinical trials, even outperforming lifestyle interventions [11,12]. The TZD class of drugs act as agonists of the transcription factor peroxisome-proliferator-activated receptor gamma (PPARγ) [13]. PPARγ is important for adipogenesis [14] and also displays anti-inflammatory activity [15]. Thus, the therapeutic actions of TZDs are likely to result from a multitude of regulatory actions at the gene expression level via PPARγ activation.

In the present study, we investigated whether the TZD pioglitazone could reverse existing hyperglycemia in *db/db* mice, a genetic model of obesity and T2D [16]. We found that pioglitazone rapidly restored glycemia to levels observed in non-diabetic lean littermate control mice. This complete restoration in blood glucose concentration was associated with shifts in respiratory quotient to reflect greater whole-body carbohydrate oxidation, an observation consistent with increased glucose utilization and improved insulin sensitivity. Circulating insulin also returned to the amounts observed in lean mice while adiponectin, an insulin-sensitizing hormone, was markedly increased. Markers of browning were present in white adipose tissue of *db/db* mice receiving pioglitazone. We also found that this expression pattern was recapitulated in the femoral depot of human white adipose tissue from subjects given pioglitazone. Strikingly, pioglitazone-enhanced insulin gene expression in isolated pancreatic islets, with reductions in the de-differentiation marker Aldh1a3. In pancreatic sections, Aldh1a3 protein was decreased in mice receiving pioglitazone concomitant with increased abundance of the transcription factor Nkx6.1, a marker of mature β-cells. Therefore, we conclude that oral administration of pioglitazone in a mouse model of obesity and T2D restores several key markers of metabolic health.

## 2. Materials and Methods

### 2.1. Experimental Animals

Male C57BL/6J (Jax number 000664), *db/+* and *db/db* mice (B6.BKS(D)-*Lepr^db^*/J; Jax number 000697) were purchased from the Jackson Laboratory (Bar Harbor, ME, USA) at seven weeks of age. All animals were allowed to acclimate to the Pennington Biomedical Research Center or University of Tennessee Medical Center facilities for at least seven days to allow for normalization of physiological parameters following transport [17]. During the acclimation period, animals were given ad libitum access to Teklad 8640 Rodent Diet (Envigo, Indianapolis, IN, USA) and water. Prior to beginning each study, *db/db* mice were randomized into two dietary groups and fed Teklad 8640 Rodent Diet (supplemented or not with pioglitazone) after stratification based on body weight and blood glucose to avoid any significant differences between groups at baseline. Pioglitazone hydrochloride was purchased from Sigma Aldrich (St. Louis, MO, USA; Cat # E6910) and blended into Teklad 8640 Rodent Diet at a concentration of 105 mg/kg. The dose of PIO in the food based on the 105 mg/kg diet provides approximately 15 mg/kg per day per mouse; this is less than or very near to what has been reported for other studies [18]. Animals were placed on the control (CON) or pioglitazone-supplemented (PIO) diets for 11 to 28 days.

Three cohorts of mice were required to complete the studies described herein. For cohort 1, non-fasting blood glucose and body mass were measured on study days 0, 4, 7, 11, 14, 18, 21, 25, and 28. On day 28, following a 4 h fast, animals were sacrificed by CO_2_ asphyxiation and cervical dislocation. Blood was collected by cardiac puncture and the serum fraction was subsequently extracted. Fat depots were snap frozen in liquid nitrogen. Pancreata were perfused and islets were isolated using our previously published protocol [19]. For cohort 2, measurements of energy expenditure, respiratory quotient, activity, and caloric intake were conducted using Promethion metabolic cages (Sable Systems, North Las Vegas, NV, USA). One week before administering diets, *db/db* mice were moved from their home cages to single-housed metabolic training cages to allow for acclimation. On study day 0, animals were moved to the Promethion cages and the dietary protocol was initiated at the start of the metabolic cage measurements. On day 7, animals were removed from the metabolic cages and returned to their home cages. Non-fasting blood glucose, body mass, and body composition were thus assessed in this cohort on study days 0, 7, 14, and 28. Measurements of body composition (fat, lean, and fluid mass) were made by NMR using a Bruker Minispec LF110 Time-Domain NMR system. Cohort 3 used lean mice given either control or pioglitazone-supplemented diets. Insulin tolerance was measured using i.p. injection of Humulin R at 1 U/kg body weight after a 4 h fast. Upon completion of cohorts 2 and 3, animals were sacrificed by CO_2_ asphyxiation and decapitation following a 4 h fast. Trunk blood was collected for serum extraction. Fat depots were snap frozen in liquid nitrogen. Pancreata were fixed in 10% neutral-buffered formalin (NBF). The number of animals used is stated in the figure legend. All animal procedures described herein were approved by the Institutional Care and Use Committees of Pennington Biomedical Research Center (IACUC protocol # 1021; approved 05/02/2018) and University of Tennessee Health Science Center (IACUC protocol # 2171; approved 02/26/2016).

### 2.2. Pancreas Immunohistochemistry 

Following fixation in 10% NBF for 24–48 h, pancreatic tissue was embedded in paraffin, sectioned, stained, and analyzed as previously described [20,21]. Primary antibodies used were as follows: guinea pig anti-insulin (Invitrogen, Grand Island, NY, USA; #18-0067; 1:800), glucagon (Cell Signaling Technology, Danvers, MA, USA; #2760; 1:300), Nkx6.1 (Developmental Studies Hybridoma Bank, Iowa City, IA, USA; #F55A12; 1:100); and Aldh1a3 (Novus Biologicals, Centennial, CO, USA; #NBP2-15339; 1:100).

### 2.3. Pioglitazone-Treated Human Study Participants and RNA Isolation from Human Adipose Tissue

#### 2.3.1. Study Participant Characteristics

The Apple & Pear trial (“Cellular Dynamics of Subcutaneous Fat Distribution in Obese Women”; ClinicalTrials.gov ID- NCT01748994) was a randomized, double-blind, placebo-controlled, parallel-arm trial conducted at Pennington Biomedical Research Center (PBRC). Details of the study design have been reported [22]. Briefly, healthy women, with overweight or obesity, who were 18–40 years of age and had a body mass index (BMI) of 27–38 kg/m^2^ were recruited for this study. Participants were absent of diabetes or any major organ disease, weight stable for ≥3 months (±3.2 kg), had no significant changes in diet or physical activity in the previous month, and had no chronic use of medications to cause weight gain, weight loss, or other potential metabolic effects (e.g., glucocorticoids, adrenergic agents, and thiazolidinediones). 

After screening for eligibility, women completed baseline metabolic assessments, including adipose tissue biopsy collections, and were then randomized (1:1 allocation ratio) to consume 30 mg/day of pioglitazone (PIO group) or to a placebo group for 16 weeks. PIO (30 mg), purchased from an outside pharmacy, was repackaged into capsules by the pharmacist at PBRC, and the placebo capsules were packaged in similar capsules. The PIO and placebo were administered with visits every 4 weeks at PBRC. To monitor compliance, participants were required to return unused pills for counting. After 16 weeks, the metabolic assessments were repeated. Pennington Biomedical Research Center’s IRB approved (Protocol # 10039 -PBRC) all procedures from the originally published study [22] and all study participants gave written, informed consent. Adipose tissue biopsy samples used in this study were de-identified prior to RNA isolation outlined below.

#### 2.3.2. Adipose Tissue Biopsies and RNA Isolation

Adipose tissue biopsies were collected with the Bergstrom and the Mercedes lipoaspirate techniques under sterile conditions and local anesthesia at baseline and post-intervention. Samples were taken from the subcutaneous abdominal region, between one- and two-thirds of the distance from the iliac spine to the umbilicus, and from the subcutaneous femoral region, on the anterior aspect of the thigh, one- to two-thirds of the distance from the superior iliac spine to the patella. The tissue was immediately frozen in liquid nitrogen and stored at −80 °C. Total RNA was extracted using the miRNeasy kit (Qiagen, Germantown, MD, USA), and the yield determined by spectrophotometry (NanoDrop Technologies, Wilmington, DE, USA). From each RNA sample, 500 ng was reverse transcribed to cDNA by using the iScript cDNA Reverse Transcription kit (Bio-Rad, Hercules, CA, USA). Real-time PCR was performed using the CFX real-time PCR system (Bio-Rad).

### 2.4. Gene Expression Analysis

Mouse epididymal (eWAT) and inguinal white adipose tissue (iWAT) depots were powdered and 50 mg aliquots were homogenized in TRIzol. Total RNA was extracted from adipose tissue and isolated islets using the RNeasy Mini RNA kit (Qiagen, Germantown, MD, USA). RNA quality and quantity was assessed using a Nanodrop spectrophotometer (NanoDrop Technologies, Wilmington, DE, USA). cDNA was generated from total RNA using the iScript cDNA synthesis kit (Bio-Rad). Relative mRNA abundance was measured by real-time RT-PCR using the iTaq Universal SYBR Green Supermix (Bio-Rad) on a CFX96 instrument (Bio-Rad). Transcript levels were normalized to the housekeeping gene Rs9 [23]. Primer pairs were designed using the Primer3Plus software and sequences are available upon request. 

### 2.5. Serum Analyses

Mouse ELISA Kits from Mercodia (Uppsala, Sweden) were used to measure serum insulin and glucagon. Corticosterone was measured using an ELISA kit (Cat number ADI-900-097) from Enzo Life Sciences (Farmingdale, NY, USA). Triacylglycerol was determined using the Triglyceride Determination Kit (Sigma Aldrich; cat. no. TR0100-1KT). Mouse HMW and Total Adiponectin ELISA kit (Cat number 47-ADPMS-E01) was from Alpco (Salem, NH, USA). Manufacturers’ recommended protocols were used for all serum measurements. 

### 2.6. Statistical Analysis

Statistical analyses were performed using GraphPad Prism 6.07 (GraphPad Software, La Jolla, CA, USA). Data were analyzed by two-tailed Student’s t-test, one-way analysis of variance (ANOVA) using a Tukey’s test for post hoc analysis, or repeated-measures ANOVA (for longitudinal measures of blood glucose and body mass). Datasets were tested for outliers using the Rout method (Q = 1%). Data are presented as the means ± SEM.

## 3. Results

### 3.1. Pioglitazone (PIO) Lowers Blood Glucose in Obese Diabetic Mice

**Figure 1:**
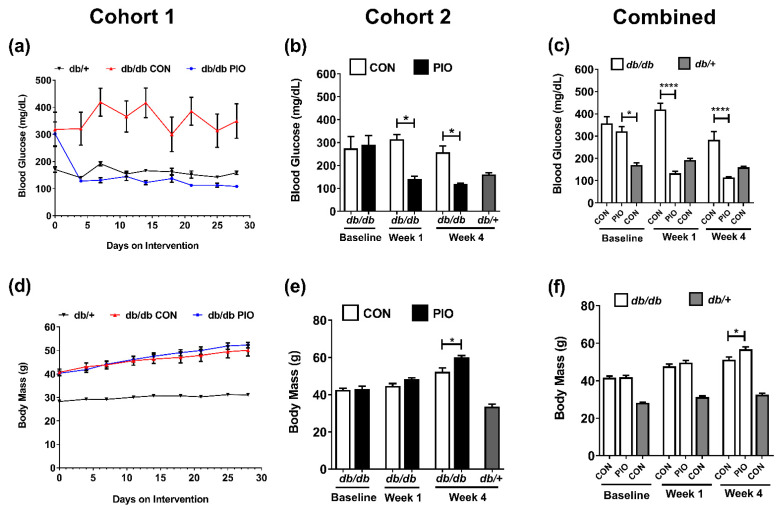
Dietary supplementation of pioglitazone (PIO) alleviates hyperglycemia in *db/db* mice. (**a**–**c**) Blood glucose in *db/+* mice on a control diet, and *db/db* mice on either a control (CON) or pioglitazone (PIO)-supplemented diet for 28 d starting at 8 weeks of age. (**d**–**f**) Body mass in *db/+* mice on a control diet, and *db/db* mice on either a CON or PIO-supplemented diet for 28 d. Data from cohort 1 are represented in Figure 1a,d (n = 6 per group), cohort 2 is shown in Figure 1b,e (n = 8 per group), and data compiled from both cohorts are shown in Figure 1c,f (n = 14 per group). Data are represented as the means ± SEM. * *p* < 0.05, **** *p* < 0.0001.

*db/db* mice are genetically obese and exhibit key features of human T2D, including insulin resistance, hyperglycemia, and alterations in islet β-cell markers [2,16,24,25,26]. We observed that oral delivery of the TZD pioglitazone (PIO), an insulin-sensitizer prescribed to patients with prediabetes or existing T2D [11,27,28], counteracted these pathological metabolic outcomes. Blood glucose in obese *db/db* mice (mean = 357 mg/dL at baseline) was restored to concentrations observed in lean littermate (*db*/+) controls four days after PIO administration (Figure 1a–c). These data are consistent with previous observations [29]. Pioglitazone has no effect on blood glucose, circulating insulin, or insulin tolerance in lean normoglycemic mice (Supplementary Figure S1). We did note a modest but significant increase in insulin positive area, islet fraction, and major and minor axis length in the islets of lean mice receiving pioglitazone. However, circulating insulin was not changed (Supplementary Figure S1). Because we were interested in the impact of pioglitazone during the obese, hyperglycemic state, a condition for which it is prescribed to humans, we did not study lean normoglycemic mice on pioglitazone any further. 

Blood glucose remained in the normal range in PIO-treated *db/db* mice for the duration of the 28 day dietary study with no evidence of hypoglycemia (Figure 1a–c). Compared to lean control *db*/+ mice, *db/db* mice displayed a body mass of 41.7 g (obese) versus 28.3 g (lean *db*/+; Figure 1d). PIO-treated *db/db* mice did not differ in body mass after one week on the drug (Figure 1e). However, there was a significant increase in body mass at the end of the 4 week study between *db/db* mice on PIO versus *db/db* mice consuming the control diet (56.7 g vs. 51.3 g body mass; Figure 1f; week 4). 

### 3.2. Four Weeks of PIO Therapy Increases Fat and Fluid Mass in db/db Mice

**Figure 2:**
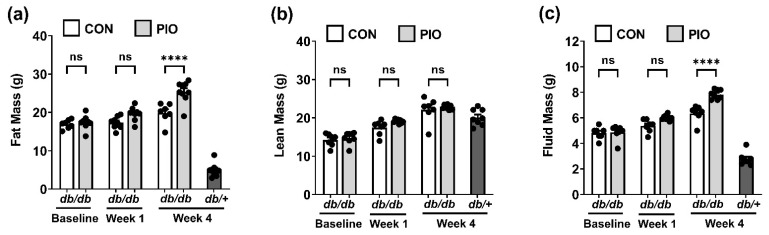
Four week dietary supplementation of PIO increases fat and fluid mass in *db/db* mice. (**a**) Fat mass, (**b**) lean mass, and (**c**) fluid mass in *db/+* mice on a control diet, and *db/db* mice on either a CON or PIO-supplemented diet for 28 d. n = 8 per group. Data are represented as the means ± SEM. **** *p* < 0.0001. ns, not significant.

At baseline, body composition was not different between the two groups of *db/db* mice (Baseline; compare white bar to light grey bar; Figure 2a–c). No significant difference in body composition was observed after one week of pioglitazone administration (Figure 2a–c); however, the mice receiving PIO display an 28% increase in fat mass after four weeks (Figure 2a) with no significant difference in lean mass (Figure 2b). Consistent with increased fat mass, there was also a 24% increase in fluid mass compared to animals receiving the control diet (Figure 2c). The lean control mouse (*db*/+) is shown for comparison (darker grey bars; Figure 2a–c). 

### 3.3. Pioglitazone Increases Respiratory Quotient (RQ) and Energy Expenditure (EE), but Does Not Alter Locomotor Activity or Food Intake

**Figure 3:**
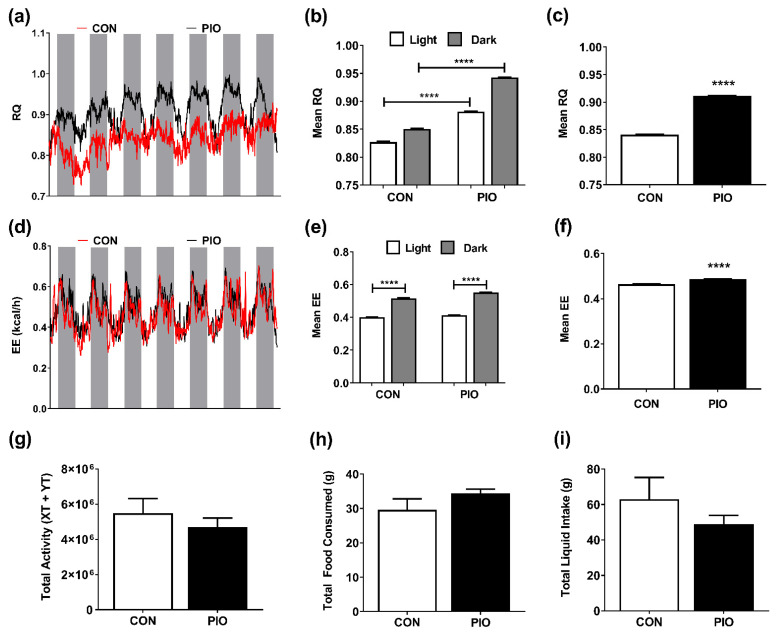
Pioglitazone increases respiratory quotient (RQ) and energy expenditure (EE), but does not impact activity or caloric intake. (**a**) RQ at daily intervals showing light (daytime; white bars) and dark (night time; grey bars) cycles, (**b**) mean RQ across light cycles, (**c**) mean RQ across all time points, (**d**) EE at daily intervals showing light (day) and dark (night) cycles, (**e**) mean EE across light cycles, (**f**) mean EE across all time points, (**g**) total activity, (**h**) total food consumed, (**i**) total liquid consumed in *db/db* mice on either a CON or PIO-supplemented diet for 7 d. n = 8 per group. Data are represented as the means ± SEM. **** *p* < 0.0001.

Because blood glucose concentrations were rapidly restored to normal values in obese mice receiving pioglitazone (Figure 1), we conducted a separate study where *db/db* mice were placed into metabolic cages and given a PIO-supplemented diet or a control diet at the start of the metabolic cage measurements. We found a rapid increase in respiratory quotient (RQ), reflecting enhanced whole-body carbohydrate utilization, in mice receiving PIO when compared to *db/db* CON mice (Figure 3a–c). Over a period of 7 days, mean RQ was significantly higher across both light (day) and dark (night) cycles in PIO-fed *db/db* mice (Figure 3a,b), with an overall increase in RQ from 0.84 to 0.91 in the PIO group relative to CON-fed animals (Figure 3c). These data, representing increased whole-body glucose utilization, are consistent with the decrease in blood glucose shown in Figure 1. 

Daily energy expenditure was similar between groups (Figure 3d) with clear differences between light and dark cycle (Figure 3e; white bar to grey bar). We noted a cumulative 4.74% increase in mean energy expenditure after seven days in PIO-supplemented *db/db* mice relative to CON animals (Figure 3f). No significant alterations in physical activity (Figure 3g), food consumption (Figure 3h), or liquid intake (Figure 3i) were observed between CON or PIO groups. Furthermore, mean sleep time was similar between dietary groups (data not shown). 

### 3.4. PIO Therapy Restores the Majority of Circulating Hormones in Obese Mice to Values Observed in Lean Controls

**Figure 4:**
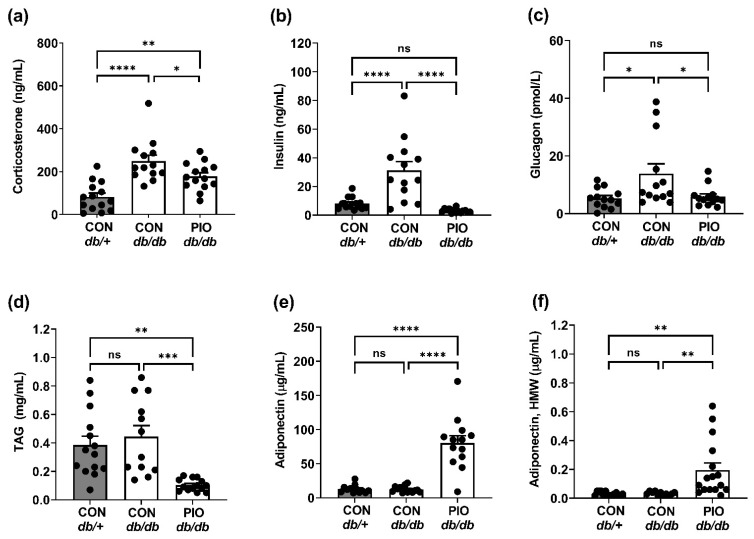
Dietary supplementation of PIO alters circulating levels of endocrine hormones, triglyceride, and adipokines in *db/db* mice. Serum levels of (**a**) corticosterone, (**b**) insulin, (**c**) glucagon, (**d**) TAG, (**e**) adiponectin, and (**f**) high-molecular-weight (HMW) adiponectin, in *db/+* mice on a CON diet (gray bars) and *db/db* mice on either a CON or PIO-supplemented diet for 28 d (white bars). n = 8 per group. Data are represented as the means ± SEM. * *p* < 0.05, ** *p* < 0.01, ***, *p* < 0.001; **** *p* < 0.0001. ns, not significant.

As shown in Figure 1, Figure 2 and Figure 3, *db/db* mice receiving PIO display normal blood glucose levels with an increase in RQ despite elevations in total body mass and fat mass. Thus, we next examined circulating hormones to investigate whether they help to explain the metabolic changes. Corticosterone promotes insulin resistance and increases blood glucose levels when elevated chronically [21,30,31]. Corticosterone quantities in serum were reduced by intervention with PIO, although not back to control levels (Figure 4a). Circulating insulin concentrations are greater in untreated *db/db* mice when compared with either *db/db* mice receiving PIO or lean control mice (Figure 4b). In addition, circulating glucagon in *db/db* mice administered PIO also returned to values observed in lean control mice (Figure 4c). The changes in circulating hormones were consistent with the reduction in blood lipid (measured as triacylglycerols; Figure 4d). Further, PIO therapy increased both total serum adiponectin (Figure 4e) and its high-molecular-weight (HMW) form (Figure 4f). Taken together, these data are consistent with improved blood glucose levels in obese mice receiving oral PIO therapy.

### 3.5. PIO Supplementation Alters Gene Expression Patterns in White Adipose Tissue from db/db Mice

**Figure 5:**
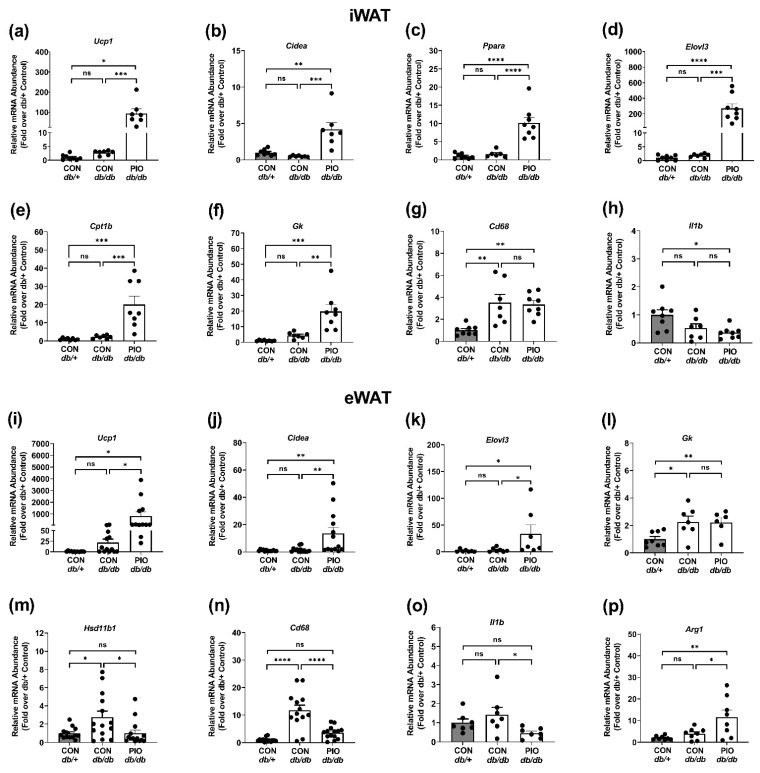
PIO alters expression of genes involved in browning, fatty acid oxidation, and inflammation in white adipose tissue of *db/db* mice. mRNA abundance of (**a**) *Ucp1*, (**b**) *Cidea*, (**c**) *Ppara*, (**d**) *Elovl3*, (**e**) *Cpt1b*, (**f**) *Gk*, (**g**) *Cd68*, and (**h**) *Il1b* in iWAT from *db/+* mice on a CON diet (gray bars) and *db/db* mice on either a CON- or PIO-supplemented diet (white bars) for 4 w. Gene expression analysis of (**i**) *Ucp1*, (**j**) *Cidea*, (**k**) *Elovl3*, (**l**) *Gk*, (**m**) *Hsd11b1*, (**n**) *Cd68*, (**o**) *Il1b*, and (**p**) *Arg1* in eWAT from *db/+* mice on a CON diet (gray bars) and *db/db* mice on either a CON- or PIO-supplemented diet (white bars) for 4 w. n = 8–14 per group. Values are represented as the means ± SEM. * *p* < 0.05, ** *p* < 0.01, *** *p* < 0.001, **** *p* < 0.0001. ns, not significant.

Exposure to TZDs typically promotes patterns of gene expression consistent with ‘browning’ of white adipose tissue [32]. Indeed, we found that *Ucp1* (Figure 5a) and *Cidea* (Figure 5b) expression were enhanced in iWAT in response to pioglitazone treatment in db/db mice. We observed no significant difference in the expression of genes typically associated with brown fat development (e.g., PRDM16) in inguinal (iWAT) or epididymal white adipose tissue (eWAT) in *db/db* mice compared to lean *db*/+ controls (data not shown). However, the genes *Ppara*, *Elovl3*, and *Cpt1b* were markedly elevated in iWAT from mice receiving pioglitazone (Figure 5c–e). These genes encode the transcription factor PPAR*α* and two enzymes involved in lipid metabolism, respectively. In addition, expression of the gene encoding glycerol kinase (*Gk*), an enzyme important for reesterification of fatty acids, was increased in response to pioglitazone in iWAT (Figure 5f). Expression of the *Cd68* gene, a marker of macrophages, was increased in iWAT from obese mice relative to lean, but was not regulated by pioglitazone (Figure 5g). The mRNA levels of *Il1b*, encoding a pro-inflammatory cytokine, was reduced in *db/db* mice receiving pioglitazone when compared with lean (*db*/+) but not when compared with untreated *db/db* mice (Figure 5h).

In comparison with iWAT, we note that the gene *Ucp1* (Figure 5i) was upregulated in eWAT of PIO-fed *db/db* mice when compared with untreated mice (52 fold in eWAT). These data are similar to what we observed in iWAT between PIO-treated *db/db* versus untreated *db/db* mice (Figure 5a; 56 fold). A similar outcome was seen with expression of the *Cidea* gene (Figure 5j; 8.4 fold increase in eWAT in PIO-exposed *db/db* versus untreated *db/db*). Additionally, similar to what was observed in iWAT, the gene encoding *Elovl3* was enhanced in eWAT from *db/db* mice receiving receiving PIO therapy (Figure 5k). By contrast, expression of *Gk* was elevated in both untreated *db/db* and *db/db* mice receiving PIO relative to lean control (*db*/+) mice (Figure 5l).

Increased availability of cortisol in adipose tissue impairs glucose and fat metabolism in individuals with metabolic syndrome and promotes insulin resistance in mice [33,34]. *Hsd11b1*, the gene that encodes the enzyme that converts inactive cortisone in humans (and corticosterone in rodents) to active cortisol, was restored to the levels seen in lean control mice (Figure 5m). Expression of *Cd68*, encoding a marker of activated macrophages [35], was markedly suppressed in eWAT by PIO exposure (Figure 5n). This was not observed in iWAT (Figure 5g). Similarly, expression of *Il1b*, a cytokine associated with pro-inflammatory macrophages, was also reduced 66% in *db/db* mice receiving PIO therapy (Figure 5o). The expression of *Arg1*, a gene associated with tissue repair and resolution of inflammation type macrophages [36], was enhanced in *db/db* mice receiving PIO (Figure 5p). Taken together, there are similarities as well as clear depot specific differences in the pioglitazone ability to regulate expression of certain targets genes in iWAT compared with eWAT.

### 3.6. Oral Pioglitazone Administration to Human Study Participants Alters White Adipose Tissue Gene Expression

Subcutaneous abdominal and femoral adipose tissues from seven women from the Apple & Pear study who had baseline (CON) and post-intervention (PIO) assessments were analyzed (26 ± 5 years; BMI 32.2 ± 3.2 kg/m^2^). The main study outcomes were previously reported [22]. These depots were chosen for their known relationships to metabolic health [37].

**Figure 6:**
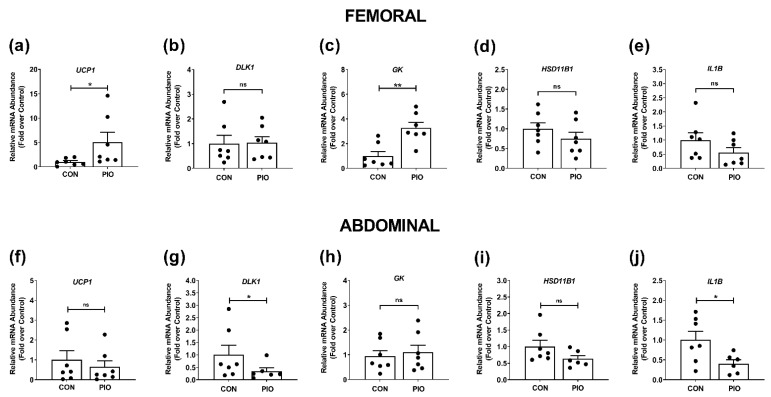
Pioglitazone reduces inflammation while also promoting markers of browning and fatty acid reesterification in white adipose tissue of human subjects. Relative mRNA abundance of (**a**) UCP1, (**b**) DLK1, (**c**) GK, (**d**) HSD11B1, and (**e**) IL1B in femoral adipose tissue from human subjects. Gene expression analysis of (**f**) UCP1, (**g**) DLK1, (**h**) GK, (**i**) HSD11B1, and (**j**) IL1B in abdominal adipose tissue from humans with the indicated conditions. n = 7 per group. Values are represented as the means ± SEM. * *p* < 0.05, ** *p* < 0.01. ns, not significant.

In femoral depots of human white adipose tissue, we found that *UCP1* expression in the group receiving pioglitazone was increased 5.1-fold over control (Figure 6a), while the expression of the *DLK1* gene was not changed under these conditions (Figure 6b). *DLK1* encodes a transmembrane protein that can be cleaved and regulates adipogenesis [38]. The glycerol kinase (*GK*) gene was upregulated 3.3-fold over control (Figure 6c) while the expression of *HSD11B1* and *IL1B* were not significantly altered by PIO (Figure 6d,e).

In contrast to the femoral depot, *UCP1* expression was not altered by PIO (Figure 6f), while *DLK1* expression was significantly reduced (Figure 6g). *GK* expression was not induced in the abdominal depot (Figure 6h). We note that *CD68* expression decreased by 51% in response to pioglitazone in the femoral depot (data not shown). The gene encoding 11β-HSD1 (*HSD11B1*), a key enzyme regulating glucocorticoid action [33], trended towards a decrease in abdominal adipose tissue with pioglitazone (*p* value = 0.11; Figure 6i). The gene encoding interleukin-1beta (*IL1B*) was reduced by 60% (Figure 6j). Note that *IL1B* was not significantly changed in the femoral depot (Figure 6e). We note that the pattern of glycerol kinase expression is similar in mouse iWAT (Figure 5f), analogous to human subcutaneous adipose tissue [39], when compared with the femoral adipose tissue in humans (Figure 6c). The abdominal depot from humans displayed patterns most comparable with mouse eWAT (compare Figure 5l with Figure 6h and Figure 5o with Figure 6j).

### 3.7. db/db Mice on a PIO-Enhanced Diet Display Increased Expression of the Insulin Genes and Decreased Expression of the Aldh1a3 Gene

**Figure 7:**
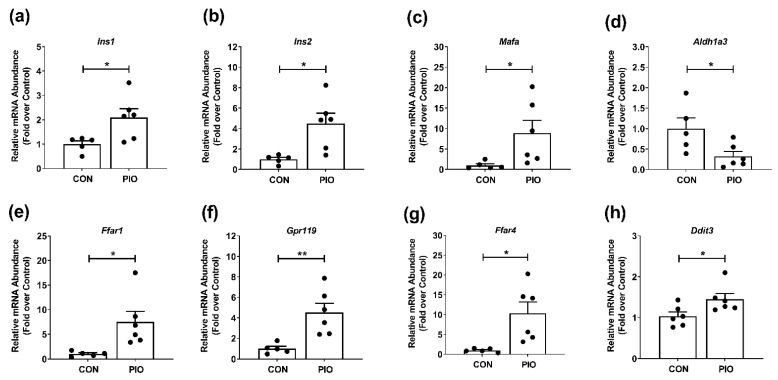
Islet gene expression reflects improvements in markers of the differentiated state in *db/db* mice receiving pioglitazone. Gene expression of (**a**) *Ins1*, (**b**) *Ins2*, (**c**) *Mafa*, (**d**) *Aldh1a3*, (**e**) *Ffar1*, (**f**) *Gpr119*, (**g**) *Ffar4*, and (**h**) *Ddit3* in islets isolated from *db/db* mice fed either a CON or PIO-supplemented diet for 28 d. n = 6 per group. Data are represented as the means ± SEM. * *p* < 0.05, ** *p* < 0.01.

Islets from humans with T2D show clear evidence of de-differentiation as measured by loss of key β-cell transcription factors (e.g., *MafA* and *Nkx6.1*) and gain of *Aldh1a3* [25,26]. The *db/db* mouse recapitulates many features of human T2D, including obesity, insulin resistance, hyperglycemia, and the aforementioned changes in markers of mature β-cells (e.g., Aldh1a3, insulin, and Nkx6.1) [16,24]. After four weeks of PIO administration, islets isolated from *db/db* mice had greater expression of both *Ins1* and *Ins2* genes (Figure 7a,b). In addition, *MafA* expression was also increased (Figure 7c). Moreover, expression of *Aldh1a3* was reduced alongside increased expression of *Ffar1* (Figure 7e), *Gpr119* (Figure 7f), and *Ffar4* (Figure 7g). We did note a mild increase in the expression of *Ddit3*, a gene linked with ER stress, in islets isolated from PIO-exposed mice (Figure 7h).

### 3.8. PIO-Supplemented Diet Restores Pancreatic Nkx6.1 Abundance and Decreases Abundance of the De-Differentiation Marker Aldh1a3 in db/db Mice

**Figure 8:**
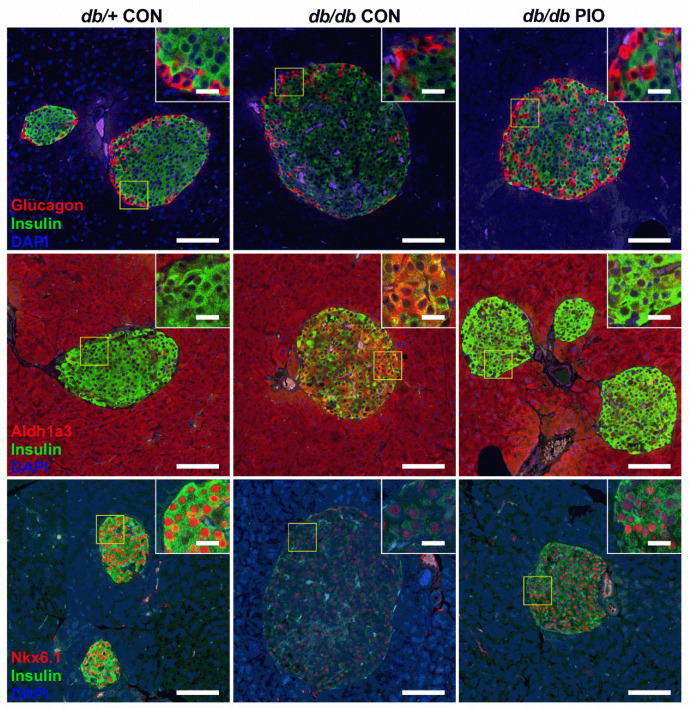
PIO-supplemented diet restores pancreatic Nkx6.1 and decreases abundance of the de-differentiation marker Aldh1a3 in *db/db* mice. Triple-fluorescence staining of fixed pancreatic tissue from *db/+* mice on a control diet (CON), or *db/db* mice on either a CON or PIO-supplemented diet for 28 d. Insulin staining shown in green and DAPI in blue. The red stain indicates glucagon (top row), Aldh1a3 (middle row), and Nkx6.1 (bottom row). Sections were stained from four animals per group and representative images were chosen from each group. Scale bars = 100 μm for large image, 20 μm for inset.

We next examined islet histology of *db/db* mice fed either control or PIO-supplemented diets as well as lean *db*/+ mice fed the control diet. Congruent to the gene expression observations in Figure 7a,b, islets from *db/db* mice given the PIO-supplemented diet displayed more intense staining of insulin (Figure 8; top row). In addition, the immunodetection of Aldh1a3 protein was reduced (Figure 8; middle row; compare middle panel with right hand panel). Finally, we found that immunoreactive Nkx6.1 was markedly enhanced in *db/db* receiving PIO when compared with *db/db* mice receiving the control diet (Figure 8; bottom row; compared middle panel with right hand panel).

## 4. Discussion

Pioglitazone is an FDA-approved PPAR*γ* agonist used to treat metabolic diseases, such as T2D [40,41,42,43]. The benefits of pioglitazone are through insulin sensitization, improved lipid metabolism, and regulation of inflammation [40,41]. In the present study, the reduction in circulating glucocorticoids (Figure 4a) and lipids (Figure 4d), as well as the rise in adiponectin (Figure 4e,f), are consistent with changes likely to reflect improvements in whole-body insulin sensitivity. Along these lines, improved insulin sensitivity was indirectly reflected by reduced serum insulin (Figure 4b) and glucagon concentration (Figure 4c) as well as by improved blood glucose levels in *db/db* mice receiving pioglitazone (Figure 1a). Importantly, we note that blood glucose levels in *db/db* mice receiving pioglitazone return to the level of lean control (*db*/+) mice without any evidence of hypoglycemia (Figure 1). In addition, pioglitazone has little to no effect on blood glucose, circulating insulin, or insulin tolerance in the lean, normoglycemic mouse (Supplementary Figure S1). We did note a slight but significant increase in insulin positive area in the islets of lean mice receiving pioglitazone, suggesting a possible direct effect of this drug to promote increased β-cell mass under these conditions. However, circulating insulin was not changed (Supplementary Figure S1).

The TZD class of drugs promotes increases in adipose tissue mass in vivo (see Figure 2a and [44]), providing a reservoir to lower lipid levels in circulation. An additional explanation for the lowered circulating lipid levels is the increased expression of adipose tissue glycerol kinase (Figure 5f), an enzyme that promotes retention and re-esterification of fatty acids in cultured adipocytes [45]. Distinct TZDs, such as rosiglitazone and ciglitazone, promote glycerol kinase expression in cultured mouse and human adipocytes as well as in adipose tissue from *ob/ob* mice [45]. Here, we extend those findings to both male *db/db* mice and female human subjects given pioglitazone (Figure 5f and Figure 6c), suggesting an important lipid lowering mechanism for the TZD class of drugs during insulin-resistant states that is relevant to both rodents and humans. We do note that while the phenotype of PIO intervention appears similar between males and females [46], it is possible that mechanisms associated with these beneficial phenotypes could be different due to differences in sex hormones. Nonetheless, our findings are congruent with improved glucose utilization as measured by the decrease in blood glucose concentration (Figure 1a) and the increase in whole-body respiratory quotient (RQ) (Figure 3a–c). The findings herein using *db/db* mice are also consistent with improved insulin sensitivity in humans [22,47]. 

The enhanced whole-body glucose utilization observed in metabolic cage studies shown in Figure 3 is also accompanied by reduced markers of islet β-cell de-differentiation and restored presence of proteins necessary to maintain mature β-cell identity (Figure 7 and Figure 8). Whether the reduction in blood lipid or blood glucose is the key variable explaining improved β-cell markers of health and maturity is unclear at present. Our best explanation is that collectively lowering serum glucose and lipids removes stress from islet β-cells, allowing them to recover. This is a postulate supported by other studies [29,48]. It is also conceivable that the effects of pioglitazone occur directly on the β-cell as well as on islet resident macrophages; these combined possibilities, along with reductions in blood glucose and blood lipid, promote increased production and storage of insulin in the islet. Interestingly, we found that pioglitazone reduces *Ald1a3* gene expression (Figure 7d) and protein abundance (Figure 8; middle row; *db/db* CON vs. *db/db* PIO) in pancreatic islets. This is important because *Aldh1a3* is a marker of islet β-cell de-differentiation in multiple different mouse models [16,20,24] and in humans [25]. These observations were also consistent with increased presence of *Ins1* and *Ins2* mRNA (Figure 7a,b) and augmented immunoreactive insulin and Nkx6.1 proteins (Figure 8).

Pioglitazone has partial PPAR*α* agonist activity [49], which may be one reason why this TZD is effective in the present study while rosiglitazone was unable to suppress Aldh1a3 expression in mouse islets in a previous study [50]. This new finding may add to the possibility of pioglitazone having underappreciated properties for treating diseases associated with obesity and insulin resistance. Our findings also provide additional pre-clinical metabolic information to aid in understanding the therapeutic potential of pioglitazone when compared with other drugs in the TZD category [42,51]. The comparison with tissues from humans in the present study support the conclusions drawn in the pre-clinical model.

Indeed, we observed that gene expression markers typically associated with brown adipose tissue (e.g., *UCP-1*) were upregulated in both mouse (Figure 5a,i) and the femoral (Figure 6a), but not the abdominal human adipose tissue (Figure 6f). In addition, there is a reduction in abdominal (Figure 6g), but not femoral *DLK1/Pref-1* (Figure 6b) in adipose tissue from humans given pioglitazone. An important observation was the enhanced expression of glycerol kinase (*Gk*) in both mouse iWAT (Figure 5f) and human femoral adipose tissue (Figure 6c). These findings are consistent with redistribution of lipid to subcutaneous adipose tissue and overall increases in BMI in response to TZD therapy [46]. Thus, pioglitazone promotes expansion of, and lipid storage within, specific adipose tissue depots as needed to decrease lipid accumulation in lean tissues and reduce circulating fatty acids. These outcomes likely arise, at least in part, through enhancing the re-esterification of fatty acids within specific adipose tissue depots in mice and humans with glycerol kinase as a key component of the mechanism (present data and [45]). Finally, we observed a reduction in IL-1β gene expression in both mouse eWAT (Figure 5o) and in human abdominal adipose tissue (Figure 6j) in response to pioglitazone. While it would be reasonable to speculate that expression of each of these genes correlates (either positively or negatively) with significant improvements in metabolic health, further in-depth studies are required to provide conclusive statistical evidence. In summary, the present data, and new evidence that pioglitazone does not have the cardiovascular risks that are observed with other TZDs [42], make it clear that pioglitazone has likely been undervalued as a practical therapeutic option for conditions associated with obesity, insulin resistance, and hyperglycemia.

## Data Availability

The data presented in this study are available upon reasonable request by contacting the corresponding authors.

## Supplementary Materials

The following are available online at https://www.mdpi.com/article/10.3390/biomedicines9091189/s1, Figure S1: Pioglitazone increases insulin positive area but does not alter circulating glucose or insulin concentrations in lean normoglycemic mice.
