# Supplementary material for: Pioglitazone Reverses Markers of Islet Beta-Cell De-Differentiation in db/db Mice While Modulating Expression of Genes Controlling Inflammation and Browning in White Adipose Tissue from Insulin-Resistant Mice and Humans
Source: Biomedicines. 2021 Sep 10;9(9):1189. doi: 10.3390/biomedicines9091189 (PMC8470788; doi:10.3390/biomedicines9091189)
Supplement: Supplementary file 1 [file biomedicines-09-01189-s001.zip › biomedicines-1294056-supplementary.pdf]

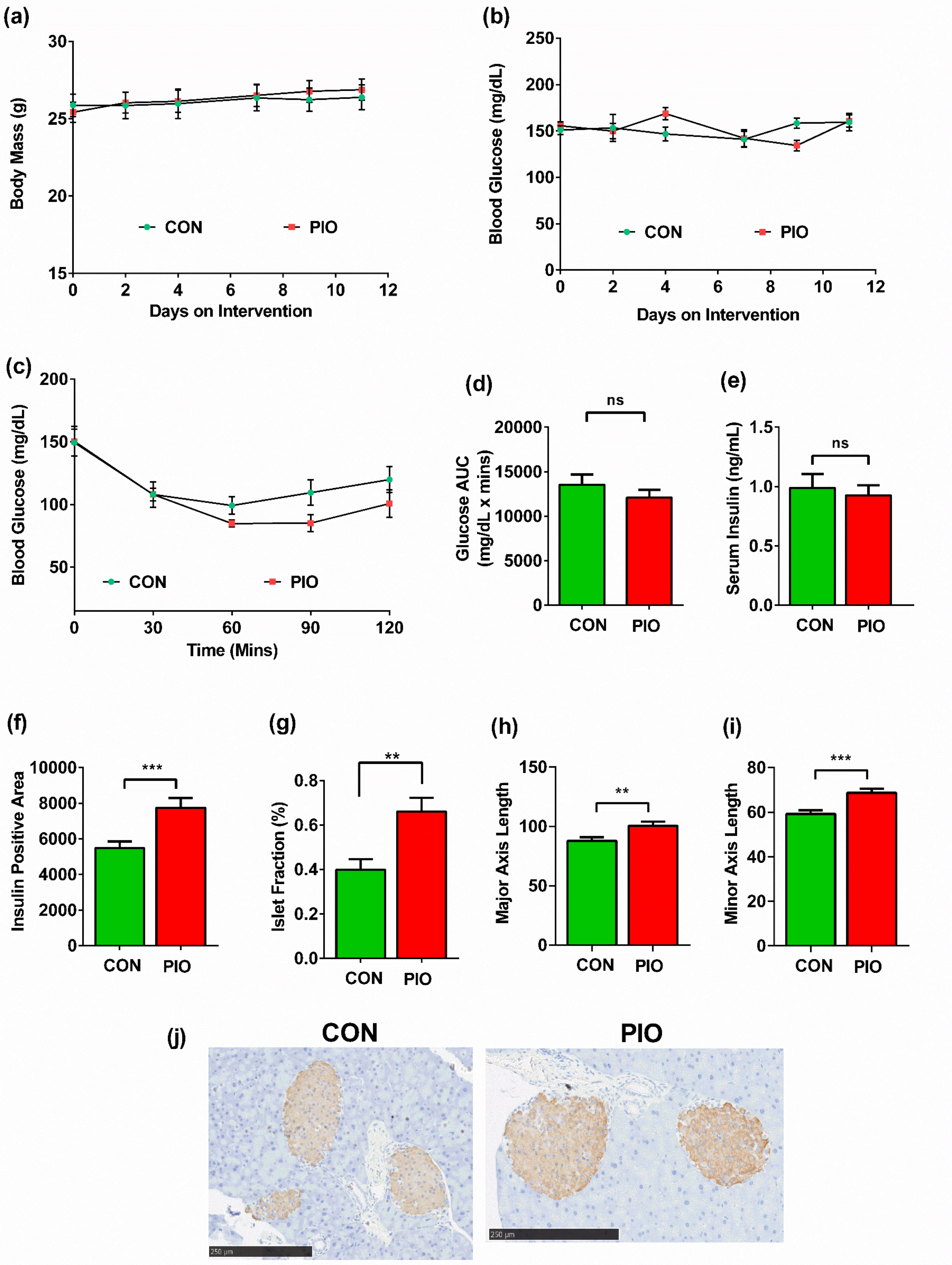

(a)
Body Mass (g)
Days on Intervention
CON
PIO
(b)
Blood Glucose (mg/dL)
Days on Intervention
CON
PIO
(c)
Blood Glucose (mg/dL)
Time (Mins)
CON
PIO
(d)
Glucose AUC (mg/dL x mins)
ns
CON
PIO
(e)
Serum Insulin (ng/mL)
ns
CON
PIO
(f)
Insulin Positive Area
***
CON
PIO
(g)
Islet Fraction (%)
**
CON
PIO
(h)
Major Axis Length
**
CON
PIO
(i)
Minor Axis Length
***
CON
PIO
(j)
CON
PIO
250 µm
250 µm

Supplementary Figure S1. **Pioglitazone increases insulin positive area but does not alter circulating glucose or insulin concentrations in lean normoglycemic mice**. Body mass (a) and blood glucose (b) are not altered by 11 days of oral pioglitazone intervention. Insulin tolerance test (c) and glucose area under the curve (Glucose AUC) analysis (d) reveals no change in insulin sensitivity in lean mice administered pioglitazone. Circulating insulin (e) is also unchanged. Insulin positive area (f), islet fraction (g), major axis length (h), minor axis length (i), and representative images (j) were collected using formalin-fixed paraffin-embedded pancreatic tissue.

**,$p < 0.01$; ***, $p < 0.001$; n.s., not significant.
